# Supplementary material for: Optical Hydrogen Sensing Properties of e-Beam WO3 Films Decorated with Gold Nanoparticles
Source: Sensors (Basel). 2023 Feb 9;23(4):1936. doi: 10.3390/s23041936 (PMC9958985; doi:10.3390/s23041936)

Supporting Information

Optical Hydrogen Sensing Properties of e-Beam WO_3 Films Decorated with Gold Nanoparticles

Elena Colusso [1,†], Michele Rigon [1,†], Alain Jody Corso [2], Maria Guglielmina Pelizzo [3] and Alessandro Martucci [1,2,*]

[1] Department of Industrial Engineering, University of Padova & INSTM, Via Marzolo 9, 35131 Padova, Italy
[2] CNR—Istituto di Fotonica e Nanotecnologie, Via Trasea 7, 35131 Padova, Italy
[3] Department of Information Engineering, University of Padova, Via Gradenigo 6, 35131 Padova, Italy
[*] Correspondence: alex.martucci@unipd.it
[†] These authors contributed equally to this work.

Figure S1. (a) Total transmittance and reflectance of pristine C-WO_3_500 film (no gold-covered); (b) digital photo of the sample taken directly above; (c) digital photo of the sample slightly tilted in order to highlight the reflectivity properties.

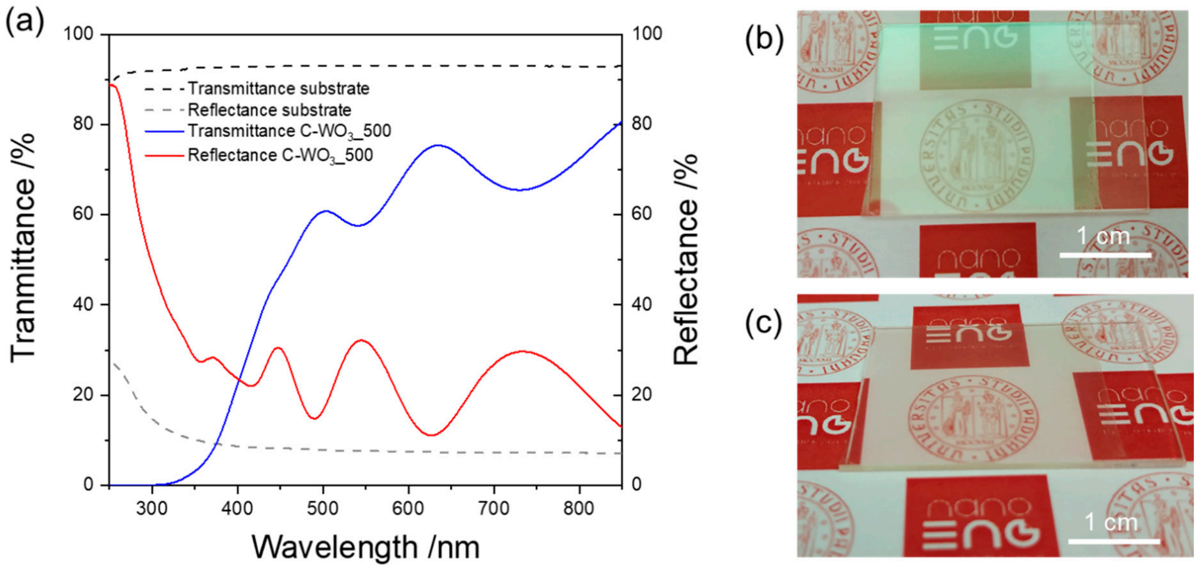

Figure S2. XRD pattern of gold covered A-WO_3 and C-WO_3_500 samples.

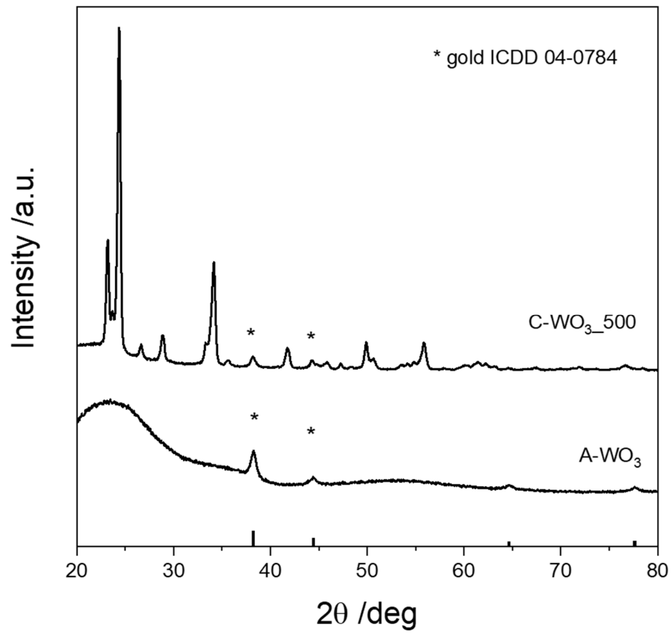

Figure S3. Au nanoparticles size distribution, mean diameter and surface coverage for C-WO$_3$_500 (a) and C-WO$_3$_300 (b).

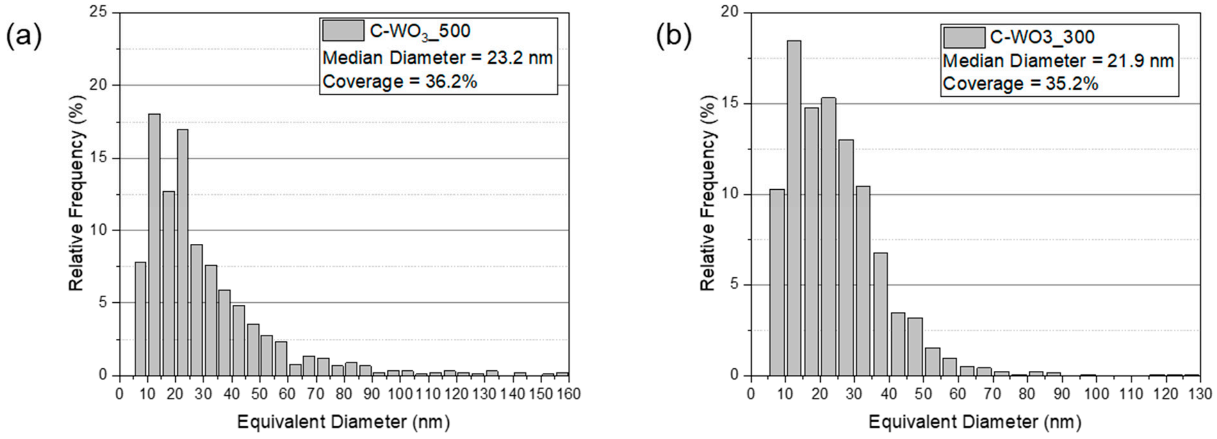

Figure S4. C-WO_3_500 characterization after sensing tests: (a) nanoparticles size distribution, mean diameter and surface coverage (a); (b) XRD pattern before and after sensing test (*w*, WO_3 peaks; *g*, Au peaks).

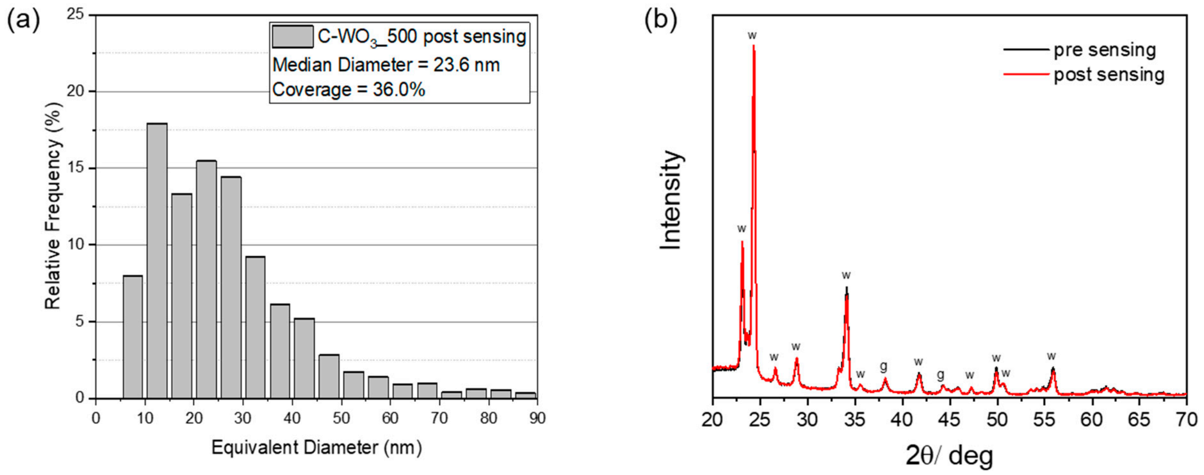

Supplement: Supplementary file 1 [file sensors-23-01936-s001.zip › sensors-2192995-supplementary(3)-highlight.pdf]
